# Bulge-Free and Homogeneous Metal Line Jet Printing with StarJet Technology

**DOI:** 10.3390/mi15060743

**Published:** 2024-05-31

**Authors:** Dániel Straubinger, Peter Koltay, Roland Zengerle, Sabrina Kartmann, Zhe Shu

**Affiliations:** 1 Hahn-Schickard, Georges-Koehler-Allee 103, D-79110 Freiburg, Germanyzhe.shu@hahn-schickard.de (Z.S.); 2 Actome GmbH, Georges-Köhler-Allee 103, D-79110 Freiburg, Germany; 3 Laboratory for MEMS Applications, IMTEK—Department of Microsystems Engineering, University of Freiburg, Georges-Koehler-Allee 103, D-79110 Freiburg, Germany

**Keywords:** liquid metal, jet, solder alloy, 3D printing, lines, printed electronics, flexible electronics, StarJet

## Abstract

The technology to jet print metal lines with precise shape fidelity on diverse substrates is gaining higher interest across multiple research fields. It finds applications in additively manufactured flexible electronics, environmentally friendly and sustainable electronics, sensor devices for medical applications, and fabricating electrodes for solar cells. This paper provides an experimental investigation to deepen insights into the non-contact printing of solder lines using StarJet technology, eliminating the need for surface activation, substrate heating, curing, or post-processing. Moreover, it employs bulk metal instead of conventional inks or pastes, leading to cost-effective production and enhanced conductivity. The effect of molten metal temperature, substrate temperature, standoff distance, and printing velocity was investigated on polymer foils (i.e., PET sheets). Robust printing parameters were derived to print uniform, bulge-free, bulk metal lines suitable for additive manufacturing applications. The applicability of the derived parameters was extended to 3D-printed PLA, TPU, PA-GF, and PETG substrates having a much higher surface roughness. Additionally, a high aspect ratio of approx. 16:1 wall structure has been demonstrated by printing multiple metal lines on top of each other. While challenges persist, this study contributes to advancing additively manufactured electronic devices, highlighting the capabilities of StarJet metal jet-printing technology.

## 1. Introduction

The process of the realisation of conductive features with additive manufacturing has gained a lot of importance and interest in recent years, since it is crucial for emerging topics such as flexible electronics [1]. The integration of electronics and sensor devices, according to the Industry 4.0 concept, also makes its way into medical and smart care applications [2,3]. However, manufacturing cost is a crucial enabler for widespread flexible and wearable electronics technology [4]. For all electronics and sensor circuits, integrated conductive structures and connections are required. For healthcare and monitoring, they are also expected to use disposable sensor elements [5], which not only presents a cost challenge, but also increases e-waste. Since reusability is often not an option due to hygiene reasons, a new interpretation of 3R (reuse, reduce, and recycle) was proposed by M. Tavakoli et al. using resilient, repairable and recyclable materials [6]. However, the environmental effect can be further reduced if the manufacturing technology is less wasteful (e.g., chemical and water consumption) and uses sustainably sourced materials and potentially biodegradable substrates [7,8,9]. The development of new, more environmentally friendly technologies would also benefit from researchers’ attention; however, the focus is instead on the lifetime and afterlife (recycling methods) of electronics [10].

There are several different technologies available for manufacturing flexible electronics. Flexible Printed Circuit Board (PCB) technology is reliable, but is a subtractive process with a fixed base material, which is not biodegradable. Additive manufacturing approaches open up new possibilities in the used substrate and conductive materials. Inkjet printing is a frequently used and well-established technique to achieve high resolution on multiple substrates. However, there are drawbacks: the cost of material (primarily silver nanoparticle based inks; considering the silver weight percentage, it can cost over EUR 5000 for a kg of conductive material [11]) is expensive, and the ink needs to be sintered at an elevated temperature, limiting the substrate compatibility [5]. Furthermore, the realised structure does not have the same conductivity as the bulk material, and the conducting area is also limited by the layer height [12,13,14,15]. Electrical component integration also requires an extra step, mainly performed with conductive adhesives (with limited shear strength and conductivity compared to soldering) [16,17]. Research is ongoing in several technological fields to realise conductive features, such as laser direct structuring (LDS) [18], magnetohydrodynamic drop-on-demand metal printing [19], or liquid-metal-based solutions [20]. However, in most cases, they are still in the research phase [21] or coming with specific bottlenecks (e.g., cannot utilise the internal volume of the structure, needs different machines for conductive or dielectric structures, very smooth surface is required, or the process is very complicated and involves chemicals and post-processing).

StarJet [22] is a metal printing technology which is capable of directly printing conductive structures to a substrate (rigid or flexible) in a non-contact (substrate distance up to a couple centimetres) manner using solder alloys or metals. The metal (raw material that can be used) is melted and superheated with a resistive heater inside a sealed reservoir. The bar form of often used solder alloys is usually in the range of EUR 50–100 per kg, and solder wire is approx. in the EUR 100–200 range per kg [23], resulting in approx. two orders of magnitude cheaper material cost compared to inkjet inks. The process does not require post-processing (i.e., curing or sintering), as the printed metal rapidly solidifies and adheres to the substrate. Since the heating is not performed on the substrate itself, there is no temperature limitation on the substrate (the amount of heat transferred is constrained by the limited volume of the printed metal). The printhead can be operated with two main modes: drop-on-demand (DoD, individual metal droplets are printed) or jet mode (continuous metal flow). The jet mode was selected as the target of this research due to the fact that continuous jet metal printing offers a quicker printing pace compared to the DoD approach, removes the possibility of omitting or adding unnecessary droplets, and could result in smaller line width (an essential feature to achieve size reduction in electronics devices). In addition, it plays a crucial role in advancing the printability of 3D metal structures.

Realising consistent line width (uniform width, no bulging, repeatable on various substrates) can be challenging; however, it is desired for improved signal integrity, reliability, and optimised resistance. Previous research has shown that spherical bulging (solder spheres formed along the line) and waviness appear at lower printing with StarJet technology, and uniform lines are achievable with increasing the printing speed [22]. However, such high speeds (up to 1000 mm/s) make it a technical challenge to manufacture more complex structures.

Directly printing jets of molten bulk metal in a non-contact manner is a novel field with limited commercially established solutions. Valcun prints aluminium at high temperatures (the bed is over 400 °C), similarly to Fused Filament Fabrication (FFF) in a contact manner, with a minimum line width of 0.8 mm [24]. PacTech GmbH demonstrated a solder ball bumper jet process, which requires a performed solder ball, which is melted with a laser and deposited to the substrate, aiming at MEMS packaging applications [25,26]. Due to the complex nature (fluid dynamics, phase change, alloy-specific properties, interaction with the substrate, and technical difficulties in realising a metal jet) of the process, researchers mainly focus on droplet printing [27,28,29]. Dimensionless parameters are often used to characterise the behaviour of the deposition [29,30,31], as summarised in the Table 1 below.

Weber number (*We*) is the ratio of inertial and surface energy [32]. The Ohnesorge number (*Oh*) describes the relationship between fluid viscosity and surface tension; the Reynolds number expresses the local inertial force to the fluid viscous force [29]. Based on these numbers, the droplet deposition dynamics can be divided into four regions of deposition: inviscid, impact-driven; inviscid capillarity-driven; highly viscous, capillarity-driven; and highly viscous, impact-driven [33]. This definition can differentiate technologies based on their kinetic mapping [29]. However, there can be significant differences between technologies based not only on the used materials (different metals and alloys), but also based on the generated droplet diameter and initial velocity of the droplet. In the case of StarJet technology, these parameters can also change with parameters such as different nozzle orifice diameters, applied printing pressures, and significantly different standoff distances (the distance between the nozzle and the substrate).

A close comparison in the challenging line uniformity is the bulging phenomenon in the case of inkjet-printed structures [34,35,36,37]. Duineveld proposed a model to describe the instability of an inkjet-printed line [38]. His results showed that a slight disturbance can grow into a bulge, for example, due to a pressure difference caused by the liquid front, if the contact angle of the liquid with the substrate is greater than the advancing contact angle at the liquid front. The bulge is then connected with a ridge to the liquid front, and after a certain length of the ridge, the contact angle can again exceed the advancing contact angle, resulting in periodic bulges. For higher substrate velocities, the bulges can also become irregular. Direct comparison is not possible because the drying behaviour of the ink is much slower than the solidification of the metal, and material properties such as surface tension can differ significantly (inkjet inks tend to have a surface tension below 100 mN/m [39,40,41], while commonly used solder alloys tend to have a surface tension around 500 mN/m [42,43,44]).

This paper aims to provide a deeper understanding of jet line printing with StarJet technology. An experimental investigation was performed to establish printing parameters resulting in uniform line widths, and to eliminate bulges along the lines when using lower printing speeds.

## 2. Materials and Methods

**Figure 1:**
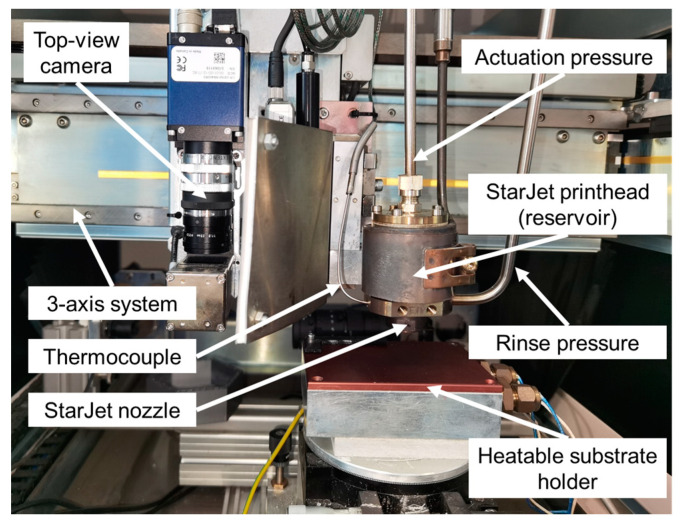
Modified Nordson Asymstek Spektrum II three-axis robotic system with integrated StarJet printhead (pressure controllers to provide accurate rinse and actuation pressure) and heatable substrate heater. Positioning on the substrate was performed using a top-view camera.

A modifiedNordson Asymtek Spectrum II 3-axis robotic system (Nordson Co., Westlake, OH, USA) with a substrate holder with built-in heating option using PID (Proportional-Integral-Derivative) control was used for the experiments, outfitted with a StarJet printhead [45], as illustrated in Figure 1. The temperature of the used substrates was monitored using a K-type thermocouple and a temperature measurement device (Voltcraft 302 K/J, Conrad Electronic SE, Hirschau, Germany). The StarJet printhead was filled with low-temperature 57Bi-42Sn-1Ag solder alloy (Ecoloy, Stannol GmbH & Co. KG, Velbert, Germany; melting range of 137–139 °C), often used in biodegradable electronics [46]. The printhead has a silicon nozzle chip, which has side channels for inert sheathing gas (referred to as rinse gas), available with different orifice diameters. The rinse gas also protects against oxidation in the nozzle, and also in close proximity. StarJet-printed AlSi112 alloy droplets exhibited almost no oxidation during the printing process [47]. The effect of rinse gas is supposedly reduced as the distance is increased, and the surface of the metal can undergo oxidation. However, this posed no obvious print quality variation in either the merging for drop-on-demand or in jet printing. The experiments provided in this paper used a nozzle chip with an orifice diameter of 44 µm. The printing can be initiated pneumatically with the actuation pressure through a valve. Both the actuation and rinse gas were nitrogen, with a pressure of 800 mbar and 860 mbar, respectively.

The jet line printing experiments were conducted on Mylar^®^ A PET (polyethylene terephthalate; DuPont Specialty Products GmbH & Co. KG, Wiesbaden, Germany) sheets that had a thickness of 125 µm and featured a smooth surface as well as high optical transparency. The sheet did not have any coating (e.g., hydrophilic or hydrophobic) to avoid alteration in the wetting angle. These thin coatings affect surface wettability, and were investigated for inkjet printing [48]. The experimental analysis consisted of crucial printing parameters, including printing velocity, standoff distance, reservoir temperature, and substrate temperature. The printing velocity (v, moving speed of printhead) was in the range of 30–120 mm/s with 5 mm/s step size. Different standoff distances (d) were investigated from 6 mm to 30 mm with a step size of 2 mm. Elevated reservoir temperature (T_res_) was analysed with different standoff distances (6–20 mm). The effect of substrate temperature was investigated in the range of 45–110 °C with a 5 °C step size (for the other test cases, the substrate was at room temperature, 20 °C). The test cases are summarised in Table 2.

High-speed video recording was used to analyse the initial starting of the solder jet and the line printing using a Phantom Miro 310 high-speed camera (Vision Research, Wayne, NJ, USA) and a Canon 5D camera (Canon Inc., Tokyo, Japan). For the high-framerate recording of jet formation, a 60 µm orifice diameter was used for increased visibility. It is essential to gain knowledge on the jet fluid dynamics for the technology, since it can reveal problems such as jet breakup or pinching behaviour in the case of continuous inkjet printing [49,50]. A qualitative estimation of the *We*, *Oh*, and *Re* numbers are provided based on measuring average weight of printed solder amounts with a 350 ms valve opening time using a nozzle chip having an orifice diameter of 44 µm. A Sartorius ME36S (Sartorius AG, Göttingen, Germany) balance was used for the weight measurement of 10 prints. The characteristic length of 44 µm was used. The material parameters were estimated based on the literature data, considering the eutectic Sn-Bi alloy at 330 °C, as follows: *σ* = 0.43 [N/m], µ = 1.5 [MPa*s], and ρ = 8500 [kg/m^3^] [51]. The average jet velocity was calculated based on matching the weight to a column of jet, having the diameter of the nozzle orifice, according to Equation (1).

(1) $$V=\frac{m}{t\left(\rho {\left(\frac{D}{2}\right)}^{2}\pi \right)},$$

where *m* is the average measured weight of printed solder, and *t* is the valve opening time.

**Figure 2:**
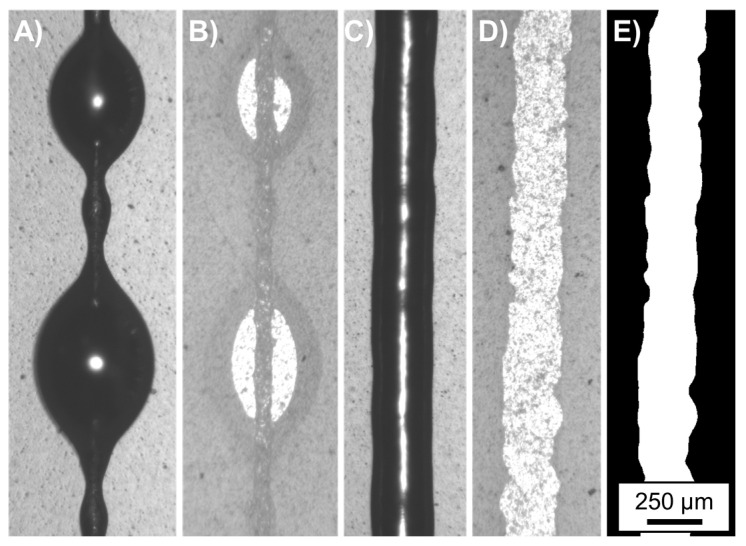
Printed Solder lines with a bulging ((**A**)—top view, (**B**)—bottom view, printed with 6 mm distance and 30 mm/s velocity) optimised bulge-free line ((**C**)—top view, (**D**)—bottom view, printed with 20 mm distance and 30 mm/s velocity), and a binary representation of the bulge-free line from the bottom view (**E**).

The transparency of the substrate material was used to image the printed solder lines at their bottom surfaces with a Zeiss Axiophot optical microscope (Carl Zeiss AG, Oberkochen, Germany). Since the printed lines have a high aspect ratio (close to 1), imaging from the top results in a shadowing effect, which can hide the spreading behaviour along the edges. Figure 2 illustrates spherical bulging (Figure 2A—top view; Figure 2B—bottom view) and an optimised, uniform line (Figure 2C—top view, Figure 2D—bottom view) and the binary representation used for evaluation (Figure 2E).

The line fidelity of bulge-free lines (since that is a desired criterion) was quantitatively analysed. In the case that no bulging was present along the lines, at least two images were taken with the microscope: one close to the starting point, and one close to the endpoint. The results were evaluated in an automated manner. The images were segmented using CVAT, an open source, machine learning-based annotation tool [52]. The usage of the tool made it possible to perform the segmentation of the lines even on low-contrast images, where more common approaches, such as thresholding and edge detection, did not deliver an acceptable result. Following segmentation, the images were binarized. Then, the width of the printed lines was measured along the images for each pixel line.

After determining parameters for bulge-free printing, further experiments were performed to utilise the results and validate the applicability on different substrates. Different rectangular substrates were fabricated using a Snapmaker 2.0 3D printer (Snapmaker, Shenzhen, China) with a nozzle size of 0.4 mm using the FFF method, which has a much higher surface roughness compared to the PET sheets used for the parameter optimisation (it is assumed that rougher surfaces make it more challenging to realise a uniform line). The substrate materials of PETG (polyethylene terephthalate glycol), TPU (thermoplastic polyurethane), PA-GF (polyamide filled with glass fibre), and PLA (polylactic acid) were used. The top surface (the last printed layer) was used for all the samples for the experiment. The infill for the polymer printing was set to a 45° setting, defining the nozzle movement direction inside each layer. Five metal lines were printed with the same printing parameters (determined from the results of Test Case A-D) on the different substrates, each having a length of 50 mm. The direction of the solder printing was parallel to the sides, resulting in a 45° and a 135° relative angle to the polymer-nozzle direction. Due to the lack of transparency, analysis of the samples was only performed from the top view. The surface profile was analysed using a Toolinspect Modell S (confovis GmbH, Jena, Germany) optical 3D measurement system with Structured Illumination Microscopy (SIM) and Focus Variation (FV) mode. To lay down a foundation for metal-on-metal printing, the same printing profile was used to print 16 lines on top of each other (which can be considered to be printing on a solder substrate), realising a self-standing wall structure. A substrate of glass plate, coated with standard printed circuit board coating lacquer, providing an excellent adhesion for the metal, was used.

## 3. Results

### 3.1. Molten Metal Jet Generation

**Figure 3:**
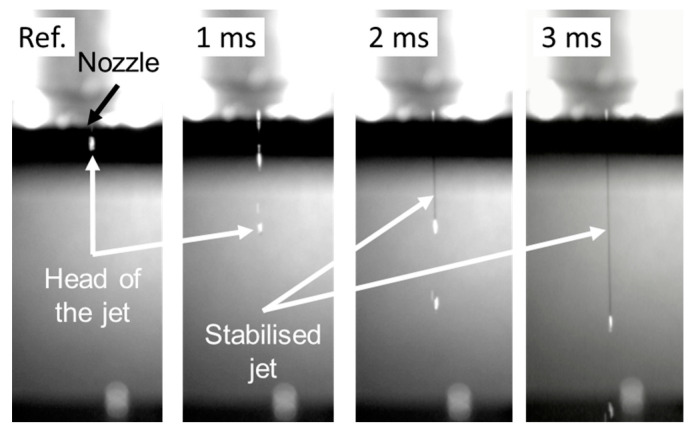
High-framerate recording of starting jet printing. A nozzle orifice of 60 µm was used for better visibility in jet behaviour (frames are 1 ms apart).

The jet formation is a critical point of the printing process of conductive line structures onto the substrates. The flow rate of the metal is dependent on the orifice diameter and the rinse and actuation pressure applied. The jet has a diameter similar to the indicated diameter of the nozzle orifice. The co-flowing rinse gas with the metal jet supposedly plays a role in the stabilisation of the jet and in preventing Rayleigh break-up [53]. Turbulence or disturbance in the jet can hinder the performance of the line formation. In particular, if the jet breaks up into separate parts during the printing process, it could result in a discontinuous structure. An example of the starting of the jet printing process captured with high-speed recording is presented in Figure 3.

The superior directional stability of the jet is attributed to the star-shaped nozzle chip compared to round nozzle orifices [53]. If the relation between the actuation and rinse pressure is not appropriately set, circular turbulence can appear, resulting in waviness on the printed line, which gets further amplified with the increasing standoff distance. However, optimised values are available for different nozzle orifice diameters, where this behaviour is eliminated, and the jet has high directional stability. The jet dynamics were similar for the different recordings. In some instances, the separation of the initial (head) part of the jet is observable. After that, the jet stabilises, and no further separation is observed during printing. It is also important to highlight that no satellite droplets are present. For most applications, the separated head part is no liability, since it remelts with the stabilised jet as it reaches the substrates. If the application is more demanding, priming the printhead (by initiating a printing sequence outside the printing area) or masking the structure is a suitable solution to eliminate this effect.

The dimensionless *We*, *Re*, and *Oh* numbers were estimated (material parameters are temperature dependent, and the sudden temperature change is not considered) according to the equations in Table 2. The average measured weight of the 350 ms solder printing was 7.64 mg. Using Equation (1), the jet velocity resulted in 1.69 m/s. The resulting values are as follows: *We* ≈ 2.48, *Re* ≈ 0.42, and *Oh* ≈ 3.74. The low Reynolds number puts the flow to a laminar region. The *We* and *Oh* number results in the highly viscous, impact-driven region; however, it is very close to the boundary of the four regions.

### 3.2. Printing Speed and Spherical Bulging (TC-A)

**Figure 4:**
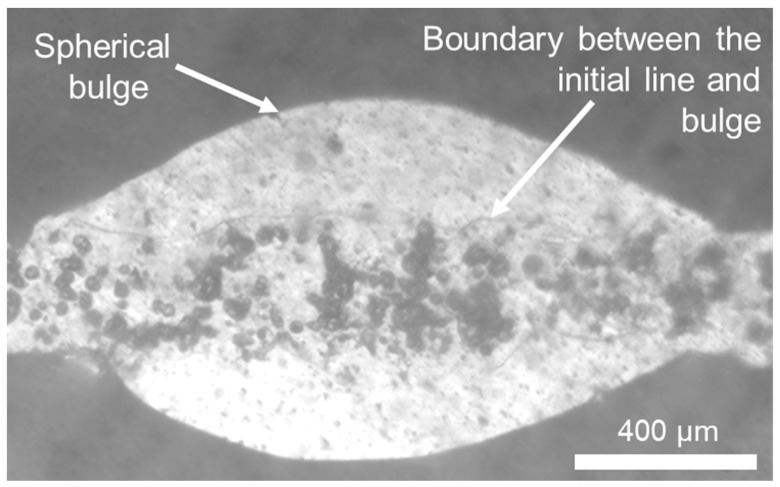
Optical microscope image of the bottom surface of a solder line with noticeable bulging printed onto a PET foil.

This study is centred on the realm of low-velocity printing, specifically targeting velocities below 120 mm/s, enabling the direct fabrication of intricate structures. While higher velocities can yield thinner, bulge-free lines, they typically necessitate velocities approximately an order of magnitude greater, approximately 900 mm/s [22]. This puts a constraint on applicability, mainly when abrupt changes in printing direction are present. The standoff distance for TC-A was fixed at 12 mm for each different printing speed. Even though the printing velocity was increased up to four times its initial value (up to 120 mm/s), the bulging was consistently present for each line. However, a slight decrease was observable in the ridges between the bulges as the printing speed was increased. An example bottom surface of a spherical bulge is illustrated in Figure 4.

The analysis of the bulge’s bottom surface revealed significantly different surfaces with a clear boundary between them. The line part in the middle contained several black dots appearing alongside it. On the contrary, the bulge formed along the line is characterised by a smooth surface. The reason for the formation of the black spots is still uncertain; however, it is assumed to be either a blistering effect resulting from heat from the metal, or due to air encapsulation due to the high initial impact velocity of the metal line, which was published before for the case of metal droplets [54,55].

Due to this significant difference in the nature of the bottom surface, the assumption was made and validated with video recordings showing that the bulge is grown after the liquid front had passed its location. The printed alloy is still in the liquid phase, and the bulge is growing in size while the jet liquid front has already moved forward. Results from Yang et al. indicated that even at otherwise stable printing conditions for inkjet printed lines, bulges could be formed due to pre-existing surface defects and the resulting pressure gradient and internal flow [36]. This explains the appearance of non-periodical bulging. On the contrary, in StarJet-printed lines, quite different bulging frequencies were observed in other experiments on different substrates (which also affected cooling, adhesion, surface energy, etc.). Even though the bulges do not necessarily show (perfect) periodicity in the experiments presented in this paper, no apparent scratches or surface defects were visible under the bulges. This suggests that bulging (even if not periodical) is rather dependent on printing parameters (and resulting features such as initial spreading and internal pressure) instead of surface defects. The versatility of StarJet on multiple substrates with optimised parameters is detailed in the Section “Printing on different substrates”.

### 3.3. Standoff Distance (TC-B)

**Figure 5:**
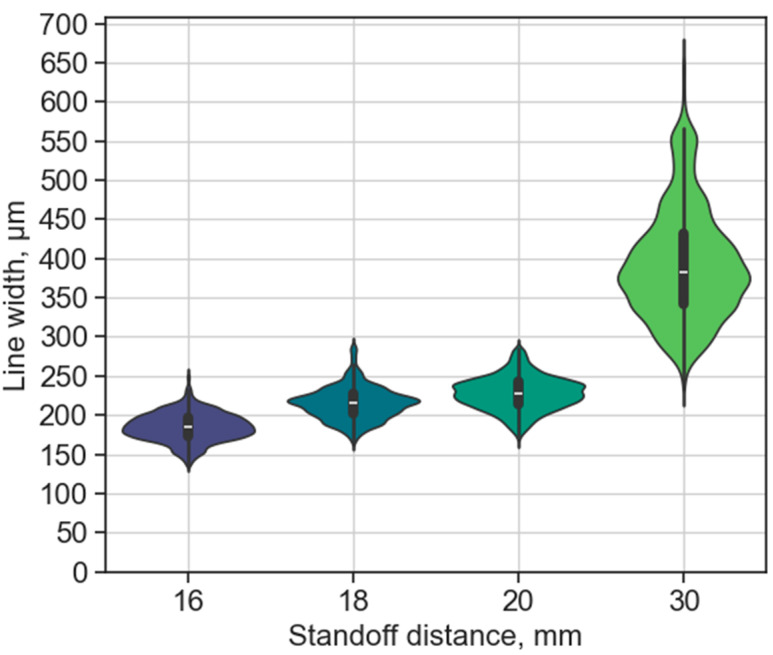
Effect of different standoff distances on line width without bulging using a 30 mm/s printing velocity. The distribution is illustrated for each dataset with a box plot inside, showing the median value with the white line.

A unique feature of StarJet printing is the variable, substantial, non-contact distance (it can go up to a couple of times 10 mm). This enables printing on uneven surfaces or flat surfaces surrounded by obstacles (e.g., soldered components, device housing) as long as their height is lower than the standoff distance. The technology can benefit from closer proximity, since it reduces the heat loss in air and oxidation (due to the rinse gas flow of nitrogen). Thus, the DoD approach is performed in most cases below a distance of 10 mm. However, the higher standoff distance and printing velocity reduce the heat transfer from the reservoir to the substrate, further extending the potentially used (heat-sensitive) substrate materials. The results showed that bulging persisted below a standoff distance of 16 mm. However, variation in line width and uniformity was present even in the bulge-free lines for distances in the range of 16 mm to 30 mm, as illustrated in Figure 5. This quantitative analysis of the printed line uniformity only consists of bulge-free printing results for all the following test cases (TC-B, TC-C, and TC-D), since that is the desired outcome for a reliable process.

**Figure 6:**
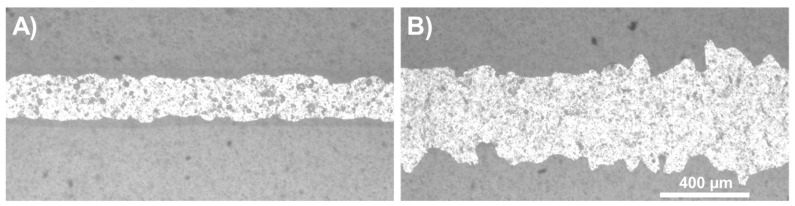
Examples of the bottom surfaces of bulge-free lines printed with a reservoir temperature of 330 °C, a printing velocity of 30 mm/s, and two standoff distances: (**A**) 16 mm; (**B**) 30 mm. The width and non-uniformity are increased in the case of the 30 mm standoff distance.

The average line width showed a slight increase as the standoff distance was increased up to 20 mm (185 µm (stdev. 18.1 µm), 216 µm (stdev. 20.8 µm), and 228 µm (stdev. 21.4 µm) for 16 mm, 18 mm, and 20 mm, respectively). The plot shows the distribution of the values for each distance and shows a box plot inside, with the median value presented. The line width is about five times higher than the nozzle orifice due to the low printing velocity (30 mm/s; the slower the printing, the higher the amount of material deposited along a specific length). The 30 mm distance was included as an edge case for the current machine setup, as it is the highest distance which can be set. This case showed a significant increase in both average line width (398 µm) and standard deviation (71.4 µm), resulting in a splashing-like behaviour, as can be seen in Figure 6 in comparison with an example of the 16 mm case.

This behaviour might be attributed to the following: as the standoff distance increases, both the length and mass of the metal fluid jet increase proportionally. The increased mass also leads to a higher kinetic energy of the fluid. The kinetic energy is converted to surface energy upon impact, thus increasing spreading [56]. A revised Weber number was proposed to consider the relative importance of initial kinetic energy, especially at lower (*We* < 30) values [57]. Additionally, the increased standoff distance allows for a more significant cooling of the molten metal before it reaches the substrate. With the increased spread on the substrate, a larger surface area is in contact, which enhances heat conduction and potentially reduces the retraction of the solder. The retraction effect (the metal is retracted after the initial splashing due to its surface tension) of the molten metal can also be dependent on the adhesion and surface energy of the substrate.

### 3.4. Reservoir Temperature (TC-C)

**Figure 7:**
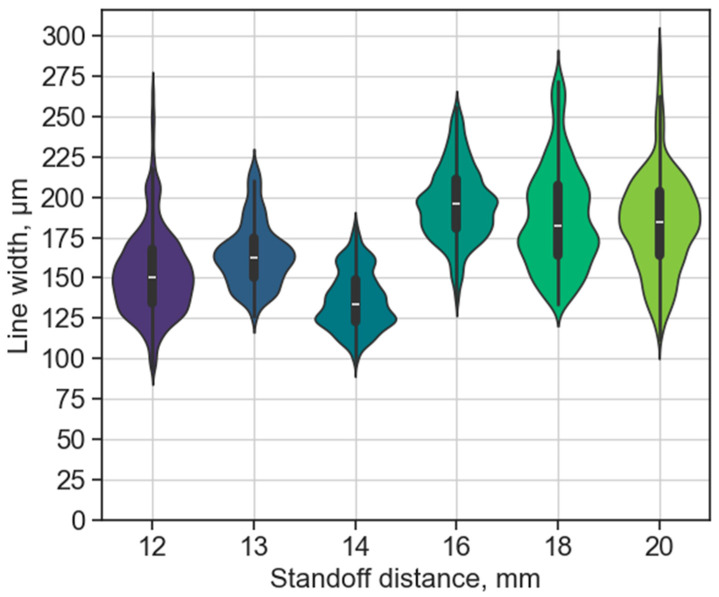
Effect of 400 °C reservoir temperature on line width and bulge-free standoff distance at different standoff distances, with a printing velocity of 30 mm/s. The distribution is illustrated for each dataset with a box plot inside, showing the median value with the white line.

**Figure 8:**
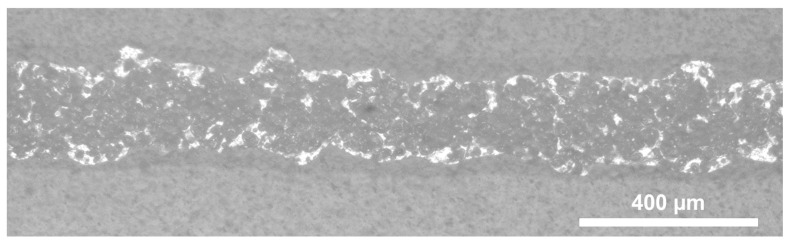
Example of the bottom surface of a printed line, using a standoff distance of 16 mm, a reservoir temperature of 400 °C, and a printing velocity of 30 mm/s, showing lower contrast and increased black spots compared to Figure 6.

In this test case, the reservoir, and thus the melted metal, was heated to an elevated temperature of 400 °C. The higher initial temperature results in lower viscosity, density, and surface tension of the Sn-Bi-Ag solder [58]. The shrinkage of the solder will be slightly higher due to the initial temperature. Furthermore, the increased thermal energy keeps the alloy longer in the liquid phase, and could also alter the interaction with the polymer substrate (e.g., degrading the material). The bulge-free line uniformity is illustrated in Figure 7, with relevant statistical values (average line width and standard deviation) in Table 3. No substrate-level degradation, such as warpage, was observed for the different printing heights. However, the bottom surface of certain lines showed an increased amount of black dots, resulting in lower contrast for the images (illustrated in Figure 8). This might be attributed to local melting and blistering of the polymer due to the localised excess heat. 

It was found that the standoff distance while keeping a bulge-free manner was reduced to 12 mm with the 70 °C increase in reservoir temperature compared to the 16 mm distance from TC-B. The average line width does not show a clear trend. The standard deviation was increased significantly in the case of the 18 mm and 20 mm cases (thus, they are less uniform), while the average line width was slightly reduced compared to TC-B. This might be attributed to the longer time to solidification, thus enhancing retraction.

### 3.5. Substrate Temperature (TC-D)

**Figure 9:**
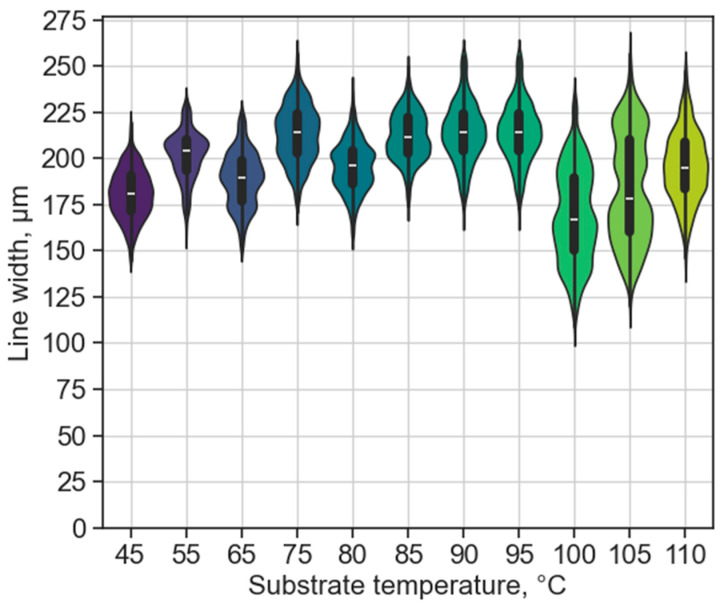
Effect of different substrate temperatures on the line width, without bulging, using a standoff distance of 20 mm and a printing velocity of 30 mm/s. The distribution is illustrated for each dataset with a box plot inside, showing the median value with the white line.

The results of elevated substrate temperatures are detailed in this section. Higher substrate temperatures could be favoured to increase the adhesion of the printed metal features of the substrate (and help with metal-to-metal connections). The results are illustrated in Figure 9 using a 20 mm standoff distance and 30 mm/s printing velocity, with relevant statistical values in Table 4.

Even though the glass transition temperature of PET sheet materials is around ≈80 °C, no warpage or material degradation was observed from the substrate heating. This also applies to the after-print condition. No evident trend is visible until a substrate temperature of 100 °C; the standard deviation is relatively low. From 100 °C, which is already over the glass transition temperature ($T_{g}$) of the PET sheet and close to the melting range of the solder alloy, the standard deviation (and thus the shape non-uniformity) of the lines was increased. Nevertheless, throughout all the samples, the used printing parameter resulted in bulge-free lines.

### 3.6. Printing on Different Rough Substrate Materials

**Figure 10:**
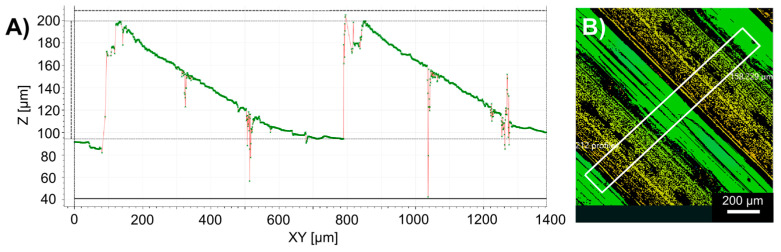
Illustration of the surface roughness measurement of PETG substrate using the Structured Illumination Microscopy (SIM) mode with a 10× objective and lateral resolution of 0.5 µm, averaged on the area marked with the white rectangle in (**B**) (profile is alongside the longer side of the rectangle). Measurement results are illustrated in (**A**), green dots representing measurement points, with red interpolation between them.

Based on the previously demonstrated test cases, the printing parameters of 20 mm distance, 330 °C reservoir temperature, and a printing speed of 30 mm/s were used, which were proven to be bulge-free in the previous experiments. A total number of 20 lines were printed on the different substrates. The printed substrates were also characterised by different surface profiles and roughness, measured for all materials. An example of the measurement is illustrated in Figure 10 for the case of PETG.

The results show a clear difference in height at the joining of the polymer lines. The total height of the roughness profile (the distance between the highest and lowest point in the Z direction along the evaluation length [59]) was approximately. 100–110 µm for PETG, 110–120 µm for PA-GF and TPU, and only around 25–30 µm for the case of PLA. These values are not unprecedented [60], but as the case of PLA suggests, there is room for optimisation in the printing parameters for the other materials. The surface profile illustrated in Figure 10 is also representative of the PA-GF and TPU cases as well. It is visible that there are missing values in between the polymer lines. This is attributed to the measurement principle of the used 3D microscope. Detecting very steep angles (i.e., in the edge between the polymer lines) is not possible; however, the highest and lowest points are accurately measurable.

**Figure 11:**
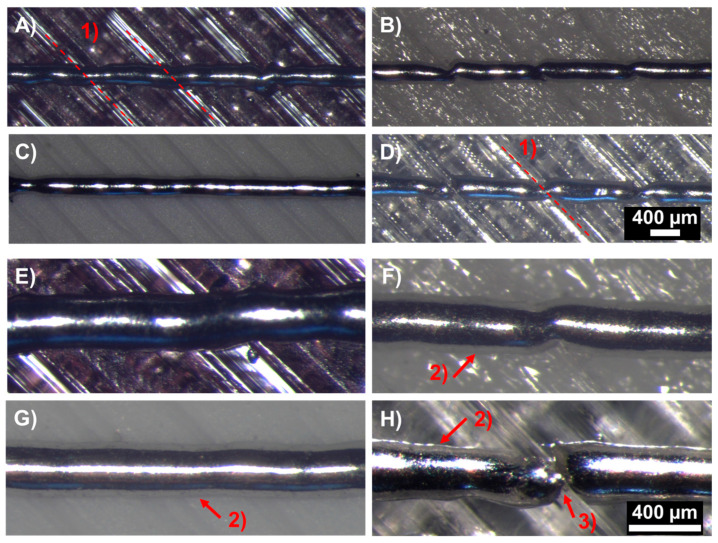
Metal lines printed on 3D-printed substrates from different polymers: (**A**,**E**)—PETG; (**B**,**F**)—PA-GF; (**C**,**G**)—PLA; (**D**,**H**)—TPU. (1) Illustrates the visible boundaries of the 3D-printed polymer routes; (2) highlights the regions on the polymer substrates around the metal lines affected by the heat of the metal; (3) shows the disturbed metal line on the TPU substrate at the edge of the 3D-printed polymer routes.

The printing results illustrate that the used material and its surface profile highly affected the printed results. An overview of the printed lines is illustrated in Figure 11.

No spherical bulging was detected for the printed lines, but slight thickening in specific locations was observable at a couple of points close to the joining locations of two polymer lines. In general, the printed lines followed the surface of the polymers. The lines on the PLA and PETG substrates were all conductive. The PA-GF sample had one defect location, resulting in one open connection out of the five lines. In the case of the TPU substrate, none of the lines were conductive. It can be seen in Figure 11F,H that the location at the edges between the polymer lines resulted in a necking behaviour, and even open connections at several locations. In those specific points, a height difference is present between the polymer lines (highlighted for some cases with (1) in the Figures), with a depth and width profile depending on the material and printing properties. This results in a longer length for the solder compared to just the horizontal difference. However, the printed volume of metal is the same for each horizontal distance; therefore, the locally increased length can disturb the solder and result in a discontinuance in the geometry, as mentioned earlier. Furthermore, the declination and inclination of the substrate affects the metal flow in the liquid state, especially if the change in height is sudden. For the PLA substrate having the smoothest surface, the effect of these boundaries is negligible and not detectable on the printed line. Even though PETG has a surface profile similar to the TPU substrate, the surface characteristics affected the printed lines less. It can be assumed that the metal–polymer interaction and the heat of the metal lines alter the surface differently for the different materials.

**Figure 12:**
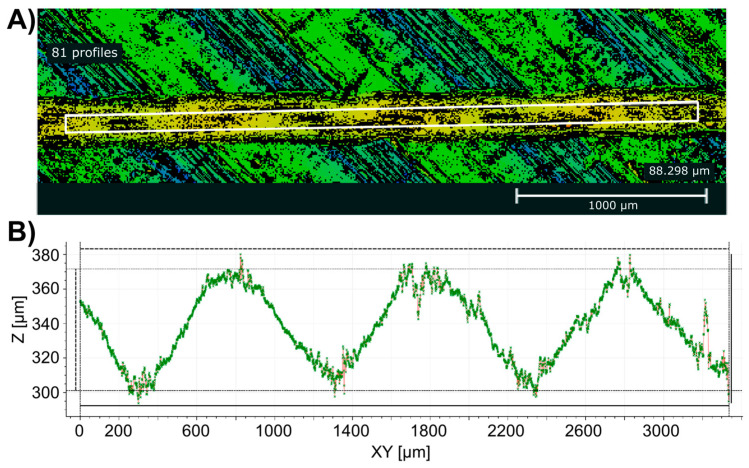
Surface profile measurement of a printed metal line on the 3D-printed polymer substrate from PETG. The measurement results in (**B**) show average height values measured in the area marked with a white rectangle in (**A**) alongside the printed metal line.

The printed lines follow the surface if they are not disrupted; an example is shown for the case of PETG in Figure 12.

The measurement shows approximately 70 µm height difference alongside the top surface of the metal line between its highest and lowest points, attributed to the roughness of the surface. It is assumed that lowering the printing speeds or using a larger nozzle orifice diameter resulting in more solder would make the technology more robust on various surfaces. These effects are worth investigating in further depth, and are planned for a separate paper.

**Figure 13:**
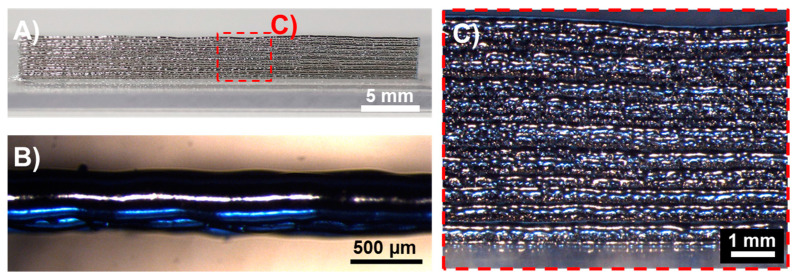
(**A**) Side overview of multiple metal lines printed on top of each other using a printing velocity of 30 mm/s, a standoff distance of 20 mm, and a reservoir temperature of 330 °C; (**B**) high magnification view from the top of the structure; (**C**) high magnification view of the side of the structure.

Printing on top of already printed metal surfaces is a crucial point towards 3D structures. The test case presented in Figure 13 illustrates the results of subsequently printing 16 metal lines on top of each other with a standoff distance of 20 mm and a printing velocity of 30 mm/s.

Results show that the printing parameters remained robust and bulge-free in the experiment. The metal lines partially melted together, forming the wall structure. However, the separate layers are still distinguishable from the side view. The top view (Figure 13B) shows excellent uniformity and line width for the multiple lines, with a promising outlook for future experiments.

## 4. Conclusions

This paper investigates the effect of crucial parameters on the uniformity of metal lines printed with StarJet technology using a nozzle orifice diameter of 44 µm and a 57Bi-42Sn-1Ag low-temperature solder alloy and PET sheet as a substrate. The effect of printing velocity and distance, substrate temperature, and elevated reservoir temperature were analysed. The experiments provide a deeper understanding of metal jet printing, specifically regarding the bulging behaviour of metal lines. Based on the results, a process window can be determined to realise bulge-free and homogeneous metal lines, which is a cornerstone of increased repeatability and reliability in metal printing. The derived robust parameter set was also evaluated on multiple FFF-printed substrate materials, characterised by a high surface roughness. The following key conclusions can be drawn:

- The bulges in the printing lines grow after the liquid front passes its location, pointing towards a probable correlation with the internal pressure of the molten metal. The bulging behaviour remained for all the test cases in the investigated printing velocity range from 30 mm/s to 120 mm/s, with a 12 mm standoff distance.
- The standoff distance is the critical parameter for line uniformity. Excessive distances can result in splashing behaviour where the lines are characterised by a higher width and non-uniformity.
- Closer standoff distance induce bulging of the lines. A uniform, bulge-free line can be realised on a PET sheet from the standoff distance of 16 mm for a reservoir temperature of 330 °C. The bulge-free standoff distance could be further reduced to 12 mm in the case of 400 °C elevated reservoir temperature.
- A standoff distance of 20 mm is recommended to realise bulge-free lines and avoid the splashing effect of the solder in the case of excessive distances.
- Substrate temperature and reservoir temperature have limited influence on line uniformity, but only induce slight linewidth deviation.
- The bulge-free metal lines can be printed from a metal jet via StarJet on rough polymer substrates from multiple materials (PETG, PA-GF, TPU, and PLA) using the optimised printing parameters from this study on PET sheets.
- Nevertheless, the elevated surface roughness posed challenges in the case of PA-GF and TPU materials, potentially causing disruptions in the metal line, such as necking or open connections at the edge between the printed polymer lines. The same parameter set also achieved a metal-on-metal structure with a high aspect ratio.

Future research is planned on optimised parameters for rough surfaces, including the scope of further understanding the material-dependent polymer–metal interaction on the substrates. The metal jet behaviour on hydrophobic surfaces is also an interest.

## Figures and Tables

**Table 1:** Dimensionless parameters describing the behaviour of metal droplet printing.

Dimensionless Parameter	Expression ^1^
Weber number	$We=\frac{\rho V^{2}D_{d}}{\sigma }$
Ohnesorge number	$Oh=\frac{\mu }{\sqrt{\rho \sigma D_{d}}}$
Reynolds number	$Re=\frac{\rho VD_{d}}{\mu }$

^1^ Where $D_{d}$ is the initial metal droplet diameter, μ is the dynamic viscosity, ρ is the density, σ is the surface tension coefficient, and V is the droplet velocity.

**Table 2:** The test cases defined for the investigation, including the parameters and their ranges.

Test Case (TC)	v [mm/s]	d [mm]	T_res_ [°C]	T_sub_ [°C]
Printing speed (A)	**35–120**	12	330	20
Standoff distance (B)	30	**6–30**	330	20
Reservoir temperature (C)	30	6–20	**400**	20
Substrate temperature (D)	30	20	330	**45–110**

Key focus parameters of the test cases are highlighted with **bold**.

**Table 3:** Statistical data of the line width from different standoff distances in Figure 7, with comparison to the results for the 330 °C reservoir temperature test case from Figure 5.

Standoff Distance [mm]	12	13	14	16	18	20
Average line width @ 330 °C [µm]	Bulging			185	216	228
Average line width @ 330 °C [µm]	Bulging			18.1	20.8	21.4
Average line width @ 400 °C [µm]	139	165	136	196	188	184
Standard deviation @ 400 °C [µm]	18	20	18	23	32	30.9

**Table 4:** Statistical data of the line width from different standoff distances in Figure 9.

Substrate Temperature [°C]	45	55	65	75	80	85	90	95	100	105	110
Average line width [µm]	181	202	189	213	196	212	214	214	169	184	196
Standard deviation [µm]	13.2	13.2	14.8	15.5	13.6	13.0	16.0	16.0	25.3	29.0	18.8

## Data Availability

The original contributions presented in the study are included in the article, further inquiries can be directed to the corresponding author.
